# Deviation of the Nasal Septum

**Published:** 1885-09

**Authors:** 


### Deviation of the Nasal Septum.

Although the introduction of the laryngoscope, nearly thirty years ago, rapidly developed a new era in the diagnosis and treatment of diseases of the larynx, it is a much shorter time since greater attention has been paid by specialists to affections of the nose and its adjacent parts. In the July number of *The American Journal of the Medical Sciences*, Dr. J. W. Gleitsmann, of New York, in an instructive paper on deviation of the nasal septum, points out the different important functions performed by the nose in the human economy, and the results of interference with these functions. The upper part of the nasal cavity, the olfactory region, presides over the sense of smell, whilst the lower one, the respiratory region, is the normal way for the air during the act of respiration. Interference with this natural channel leads to mouth-breathing with its manifold subsequent evils. When the air passes through the nose, it is not only cleansed and moistened, but it also reaches the lungs much warmer than when breathing is going on by the mouth. Nasal respiration with closed lips further exerts a negative pressure of two to four milligrammes mercury in the oral cavity, by which the tongue is drawn to the hard palate, and the muscular action, maintaining the position of the lower maxilla, greatly assisted. The nose also acts the part of a resonant chamber for the human voice, and nasal obstruction imparts to it the so-called dead character decribed in Meyer's paper on adenoid vegetations. Finally, it is due to the anatomical relations of the nose to the eye and ear that cases of catarrhal conjunctivitis, lachrymal fistula, frequently only heal when co-existing nasal affections are relieved, and that the latter are, in an overwhelming number of instances, productive of aural disease often of the severest kind. Aside from the symptoms of nasal stenosis in a greater or less degree, deviations of the septum, Dr. Gleitsmann points out, are apt to cause disfigurement of the face, and also have some relation to the bony structures of the head, which he fully explains. The pathology, etiology, symptomatology, and treatment of these deviations is fully discussed.

